# Full 3D Microwave Breast Imaging Using a Deep-Learning Technique

**DOI:** 10.3390/jimaging6080080

**Published:** 2020-08-11

**Authors:** Vahab Khoshdel, Mohammad Asefi, Ahmed Ashraf, Joe LoVetri

**Affiliations:** Department of Electrical and Computer Engineering, University of Manitoba, Winnipeg, MB R3T 5V6, Canada; masefi@151research.com (M.A.); ahmed.ashraf@umanitoba.ca (A.A.); Joe.LoVetri@umanitoba.ca (J.L.)

**Keywords:** microwave breast imaging, image reconstruction, tumor detection, convolutional neural networks, deep learning

## Abstract

A deep learning technique to enhance 3D images of the complex-valued permittivity of the breast obtained via microwave imaging is investigated. The developed technique is an extension of one created to enhance 2D images. We employ a 3D Convolutional Neural Network, based on the U-Net architecture, that takes in 3D images obtained using the Contrast-Source Inversion (CSI) method and attempts to produce the true 3D image of the permittivity. The training set consists of 3D CSI images, along with the true numerical phantom images from which the microwave scattered field utilized to create the CSI reconstructions was synthetically generated. Each numerical phantom varies with respect to the size, number, and location of tumors within the fibroglandular region. The reconstructed permittivity images produced by the proposed 3D U-Net show that the network is not only able to remove the artifacts that are typical of CSI reconstructions, but it also enhances the detectability of the tumors. We test the trained U-Net with 3D images obtained from experimentally collected microwave data as well as with images obtained synthetically. Significantly, the results illustrate that although the network was trained using only images obtained from synthetic data, it performed well with images obtained from both synthetic and experimental data. Quantitative evaluations are reported using Receiver Operating Characteristics (ROC) curves for the tumor detectability and RMS error for the enhancement of the reconstructions.

## 1. Introduction

Microwave Imaging (MWI) techniques that have been applied to the detection of breast cancer come in two forms: Radar-based techniques that attempt to detect tumors within the breast’s interior [1], and inverse-scattering based methods that attempt to reconstruct complex permittivity maps corresponding to the distribution of different breast tissues [2]. The quantitative techniques, which are of interest herein, rely on the fact that different breast tissues (e.g., skin, adipose, fibroglandular and cancerous tumors) have different dielectric properties in the microwave frequency band [3,4].

Successfully implementing the inverse-scattering approach requires that one has a good numerical electromagnetic field model for the MWI system being used to acquire scattered-field data, including the antennas and the breast, but more importantly, requires that one solves a non-linear ill-posed inverse scattering problem. This is usually accomplished using computationally expensive iterative methods where the inversion model consists of a numerical solution of an electromagnetic forward scattering problem [5]. One challenge in using MWI for breast imaging is that the breast is a high-contrast object-of-interest (OI) having complicated internal structures and this produces unique artifacts in the quantitative reconstructions of the complex-valued permittivity of the breast tissue. Both the non-linearity and the ill-posedness of the inverse scattering problem become more difficult to deal with for high contrast OIs having such complicated internal structure because they lead to multiple reflections within the OI.

The MWI technique we use in the work reported herein is the Contrast Source Inversion (CSI) method [6,7,8]. Although this is a state-of-the-art MWI technique it still succumbs to artifacts even when prior information is utilized to try to alleviate the non-linearity and ill-posedness of the problem [9,10]. Note that all MWI techniques, qualitative and quantitative alike, currently have difficulties with imaging artifacts [1,2,5,11].

Recently, there has been intense interest in the use of deep learning techniques in a broad range of applications such as natural language processing, computer vision and speech recognition [12]. In medical imaging, utilizing deep learning techniques for segmentation [13,14], as well as detection and classification [15,16,17] has been well investigated, at least for the more common modalities. Studies have shown that there is significant potential in applying deep learning techniques for the purpose of removing artifacts from biomedical images generated using some common modalities. Kang et al. proposed a deep Convolutional Neural Networks (CNNs) using directional wavelets for low dose x-ray computed tomography (CT), and results illustrate that a deep CNN using directional wavelets was more efficient in removing low dose-related CT noise [18]. Han et al. [19] and Jin et al. [20] independently proposed multi-scale residual learning networks using U-Net to remove these global streaking artifacts, In addition, domain adaptation from CT to MRI has been successfully demonstrated [21].

MWI researchers are also trying to use machine learning techniques to improve the performance of microwave imaging. For instance, researchers combined a neural network with microwave imaging to learn the forward model for a complex data-acquisition system [22]. Rekanos et al. proposed radial basis function neural network to estimate the position and size of proliferated marrow inside bone tissue with microwave imaging [23]. Le et al. tried to take the benefit of a deep neural network to enhance the constructed images [24]. Their deep neural network was trained to take microwave images created using the back-projection (BP) method as an input and have the network output a much-improved image. In fact, they tried to by-pass the use of iterative techniques for solving the full nonlinear electromagnetic inverse problem. Most recently, we have investigated utilizing deep learning techniques to improve 2D microwave imaging for the breast imaging application [25]. Researchers employing radar-based techniques have also been investigating machine learning approaches for the detection of breast lesions [26].

In this paper, we utilize a deep learning technique, based on CNNs, to enhance full 3D MWI reconstructions obtained using a 3D CSI algorithm that uses the Finite Element Method (FEM) to solve the electromagnetic forward problem [27]. The enhancement removes reconstruction artifacts and improves the accuracy of the resulting images. We utilize a 3D 10-channel U-Net architecture for the CNN where the input and output are both 3D images, and each channel corresponds to the real and imaginary parts of the complex-valued permittivity images created using five different microwave frequencies.

In Section 2 we start by providing a brief description of the CSI-based methodology that we use, as well as the numerical phantoms and MWI parameters utilized to generate training images. We also provide details of our chosen deep learning approach. In Section 3 we describe the training data set as well as the parameters used for the network training. In the following, quantitative assessment and assessment of robustness for numerical experiments are described. Section 4 provides a brief description of our experimental setup and also the result of trained CNN for the experimental data. Finally, in Section 5 we give our conclusion and explain our future work.

## 2. 3D CSI-Deep-Learning Methodology

In microwave data acquisition processes, electromagnetic fields scatter from, and propagate through, the tissue in a three-dimensional (3D) space. However, to accelerate the image reconstruction process and reduce the computational complexity, researchers are trying to represent electromagnetic waves in 3D space as a simplified 2D model. However, studies have shown that simplifying 3D problems to 2D models can increase the level of artifacts in the recovered dielectric properties [28]. Moreover, in 2D imaging when the object of interest is small, there is a chance that it place between two consecutive imaging slice, then the reconstruction algorithm would not discover the target precisely. Hence, utilizing a viable 3D microwave image reconstruction will enhance the accuracy and quality of reconstruction [29]. While iterative methods have improved dramatically over the years, providing improved resolution and accuracy of the reconstructed properties, as well as more efficient implementations, there are still many fundamental trade-offs between these three aspects due to operational, financial, and physical constraints.

Lower resolution in comparison with other modalities, as well as the many reconstruction artifacts that are related to the nonlinearity and ill-posedness of the associated inverse problem, are the main reasons that MWI is not clinically accepted yet. Although it has been shown that using accurate prior information will reduce the Root-Mean-Squared (RMS) reconstruction error over the whole image [9,10,30,31,32], artifacts and reconstruction errors near the tumor can translate to poor tumor detection results [33].

### 2.1. Microwave Imaging via Contrast Source Inversion

The first part of the proposed 3D CSI-Deep-Learning methodology consists of quantitatively generating the complex-valued permittivity images using a MWI technique. Quantitative MWI requires that one solve a non-linear ill-posed inverse scattering problem. A plethora of algorithms have been developed during the past 40 years to solve this problem. They generally involve computationally expensive iterative methods to locally minimize a specially designed functional that incorporates a numerical inversion model approximating the relevant electromagnetic phenomena of the problem [5,11]. In the past, different MWI techniques have utilized tailored optimization algorithms with various functionals. Some of the most prominent techniques have been the Distorted Born Iterative Method [34], Gauss–Newton Inversion [35], the Levenberg–Marquardt method [36] and the Contrast Source Inversion technique [6]. Innovations on these foundational algorithms have allowed improvements to the obtainable imaging accuracy and resolution, especially in the area of breast imaging, e.g., [37,38]. Being an ill-posed problem, regularization techniques are required to solve the inverse scattering problem [39,40].

As previously mentioned, to solve the electromagnetic inverse scattering problem associated with microwave breast imaging we employ the CSI method. The numerical inversion model utilized within the CSI algorithm is based on a full-vectorial 3D electromagnetic model of the MWI system that includes a quasi-resonant flat-faceted chamber [41,42]. The 3D FEM-CSI algorithm is utilized with prior information in the form of an inhomogeneous background as was done in [27]. Breast images reconstructed from both synthetic and experimental scattered-field data are utilized in this work. The experimental data is collected using the same air-based quasi-resonant imaging chamber described in [27]. Thus, the forward model for creating the synthetic data and the inversion model, both utilize a 3D finite element model of the same imaging chamber.

**Figure 1:**
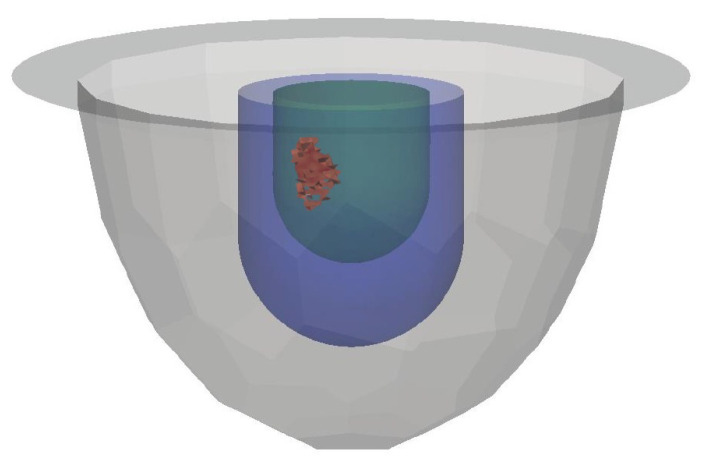
Simulated Breast Model. Gray, blue, green, and red regions represent air, fat, fibroglandular, and tumor, respectively.

We consider both synthetic and actual experimental breast phantoms with three tissue types: fat, fibroglandular and tumor. These breast phantoms are formed using a simple outer fat layer, and an interior fibroglandular region that contains one or more embedded tumors. The breast phantoms are positioned within the chamber as depicted in Figure 1.

The phantoms are interrogated using microwave energy with magnetic-field probes located on the conductive chamber walls. The same probes are used as those in receivers. As described in [42], the 24 transmitters and receivers are ϕ-polarized. Data were collected at single frequencies and for every transmitter, 23 magnetic fields were recorded at the receiver locations. Thus, 552 complex numbers (magnitude and phase) were utilized to reconstruct the breast phantom that was located within the chamber. That is, the real and imaginary parts of the complex permittivity of the breast phantom were reconstructed using the CSI algorithm.

The forward data were obtained using a 3D-FEM electromagnetic field solver. Before inverting the data using the FEM-CSI algorithm, we added 5 % noise as is usual in creating synthetic data [8]. This procedure was performed at individual frequencies and for the work considered herein, the frequency band of 1.1 GHz to 1.5 GHz was used. It has been shown that reconstruction artifacts appear at different locations of the imaging domain when different frequencies are used, whereas the tumor is typically reconstructed at approximately the same location [27]. In that work, it was shown that this feature can improve the tumor detection by using the intersection of thresholded images.

For the synthetically generated data and inversions, the permittivity was assumed to be constant over frequency. The complex permittivity values that were used are given in Table 1. For the experimental test case considered herein, the permittivities of the utilized tissue-mimicing liquids do vary with frequency (see [27] for details).

It has been shown that successful CSI reconstructions can be obtained if one introduces a fat and fibroglandular region as prior information in the CSI algorithm. This prior information is in the form of an inhomogeneous numerical background against which the contrast is defined. That is, if $\epsilon_{n}\left(r\right)$ and ϵ(r) represent the background information and the desired complex permittivity, as functions of position, then the contrast $\chi \left(r\right)=\left(\epsilon \left(r\right)-\epsilon_{n}\left(r\right)\right)/\epsilon_{n}\left(r\right)$ is one of the variables solved for in the CSI algorithm (the other variable being the contrast sources generated for each transmitter). Full details of the CSI algorithm, used in this way, are provided in [9,10].

Introducing an inhomogeneous background in this way is a form of regularizing the inverse problem, but as was already mentioned, various reconstruction artifacts are still present in the CSI-reconstructed images. These artifacts increase the false-positive and reduce the true-positive tumor detection rates. For the case of 2D imaging, it was recently shown that using a deep-learning technique ameliorates this problem [25]. This has motivated the interest in using a similar deep-learning technique to improve 3D MWI. However, in addition to artifacts, 3D MWI also suffers from the problem of producing reconstructions that do not reach the maximum permittivity values of the true phantom model. This was noted in [27] and therefore the detection threshold was based on 85% of the maximum reconstructed value. Fortunately, the tumor permittivity values are at the extreme end of the scale, so such a procedure is successful. Improving the CSI reconstructions by correcting the reconstructed permittivity values, in addition to removing artifacts is the sought after goal of using a deep learning technique.

### 2.2. Machine Learning Approach to Reconstruction

Combining the CSI technique with a deep learning approach is accomplished by learning a data-driven mapping, G, from a CSI reconstruction to the true permittivity ($G:\epsilon^{CSI}\to \epsilon^{true}$).

In this study, we learn a mapping from the real and imaginary parts of the permittivities in CSI reconstructions at several frequencies to a single real permittivity image. Thus, if the CSI complex permittivity map is an L×M×N 3D image, and reconstructions at five frequencies are utilized, then each of the learned functions maps 5×L×M×N complex domain to L×M×N real domain (e.g., $G_{R}:C^{5\times L\times M\times N}\mapsto R^{L\times M\times N}$). The complex output of CSI at the five selected frequencies can be treated as a 10-channel image. We realized this mapping through a deep neural network as follows.

**Figure 2:**
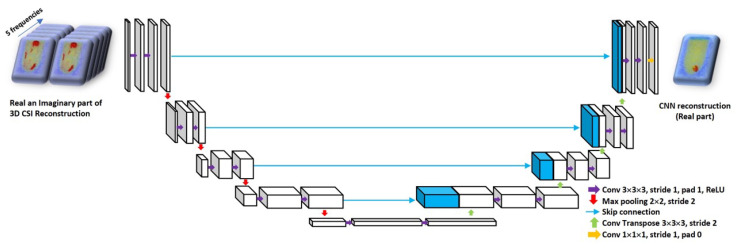
Schematic for the proposed U-Net to reconstruct the real part of permittivity. The input to the network is the 3D Contrast-Source Inversion (CSI) reconstruction, and the network is trained to output the corresponding true 3D permittivity map.

The desired mapping for our task at hand is an image-to-image transformation; there are multiple neural architectures that can implement this mapping. For instance, a naive choice could be a fully-connected single layer neural network which takes in CSI reconstruction as input and is trained to output the ground truth permittivity. However, such an architecture would be very prone to overfitting [12]. We, therefore, use a hierarchical convolutional neural network for our image-to-image transformation task. A good template for such a task is the U-Net architecture which is one of the most successful deep neural networks for image segmentation and reconstruction problems [13]. The architecture consists of successive convolutional and downsampling layers, followed by successive deconvolutional and upsampling layers. Moreover, the skip connections between the corresponding contractive and expansive layers keep the gradients from vanishing that helps in the optimization process [13,43]. To use a U-Net for reconstruction, the original objective of the U-Net is replaced with the sum of pixelwise squared reconstruction errors between the true real part of permittivity and the output of U-Net [13]. In our problem, the network input is the 3D CSI reconstructed complex images (after 500 iterations). Thus, there are two options for choosing the U-Net architecture, U-Net with complex weights and U-Net with real weights. Very few studies have been done on the training of U-Net with complex weights, although very recently Trabelsi et al. tried to train the neural network with complex weights for convolutional architectures [44]. In this paper, we decided to use a U-Net architecture having real-valued weights. A schematic representation of our architecture is shown in Figure 2. The motivation for choosing the neural network parameters (the number of convolutional layers, size and number of filters) is as follows. In a hierarchical multi-scale CNN, the effective receptive field of the convolution filters is variable at each layer, i.e., through successive sub-sampling it is possible to have a larger receptive field even by using filters of smaller kernel size [12,45]. As mentioned above, the input to our neural network is L×M×N×10; in particular, for each frequency, the dimension of our input image volume is 64×64×64 (i.e., L=M=N=64). If we start with a 3D receptive field of 3×3×3, after four layers of successive convolutions and subsampling (by a factor of 1/2), the receptive field would effectively span the entire image volume. We, therefore, use four convolutional layers with a 3D filter kernel size of 3×3×3. Since after each convolutional layer the size of the image volume is reduced, we can increase the number of filters at each successive layer to enhance the representational power of the neural network [12]. In particular, we start with 32 filters for the first layer and successively double the number of filters after each layer (number of filters after the fourth layer is 512). This defines the encoder part of the U-net i.e., the part of a neural network consisting of contractive convolutions. For the decoder part, we follow a symmetric architecture consisting of expansive convolutions [13].

## 3. Numerical Experiments

### 3.1. Datasets

While we tested our neural network on both experimental and synthetic data, for training we only used a synthetically generated dataset. The training dataset consisted of 600 numerical breast phantoms; tumors were randomly generated within the fibroglandular region of the phantom. Starting from a random initial position, tumor pixels were grown randomly until the maximum diameter reached a threshold. To have variability in the dataset, the threshold for the maximum diameter was also randomly sampled from the range: 1.1–1.5 cm. One half the dataset consisted of breast phantoms with one tumor, while the other half had phantoms with two tumors. We then employed a forward solver [8] to generate the scattered field data corresponding to the phantoms. CSI reconstructions were performed at five frequencies: 1.1, 1.2, 1.3, 1.4, and 1.5 GHz. These CSI reconstructions together with the corresponding ground-truth permittivity values for the phantoms formed our training data for the U-Net input and output respectively.

### 3.2. Network Training

All the CNNs were implemented using Python 3.6 and Keras 2.0.6 with Tensorflow backend. We used a Windows 10 computer with a Tesla P100-PCIE-12GB graphic processor and Intel(R) CPU(3.50 GHz). We used the popular Xavier initialization for the convolutional layer weights to obtain an appropriate scale [46]. We trained with a batch size of 10, for 200 epochs with Adam optimization. Four-fold cross-validation strategy has been utilized to evaluate the proposed deep neural network for all experiments. The U-net wastrained using the real and imaginary parts for five different frequencies as inputs. With 600 phantoms in our dataset, each fold in four-fold cross-validation consisted of 150 examples. For every fold, training was done using 450 cases, while the testing set consisted of the held-out 150 examples. Thus all 600 cases featured as test examples when they were not part of the training set. For the loss function, we use pixel wise mean squared error between the ground truth 3D image and the CNN 3D reconstructed image as follows:

(1) $$RMSError=\frac{1}{LMN}\sum_{x=1}^{L}\sum_{y=1}^{M}\sum_{z=1}^{N}(I_{x,y,z}^{GT}-I_{x,y,z}^{CNN}{)}^{2}$$

where $I_{x,y,z}^{CNN}$ represents a 3D image reconstructed by the CNN and $I_{x,y,z}^{GT}$ represents a 3D ground truth image.

### 3.3. Quantitative Assessment

**Figure 3:**
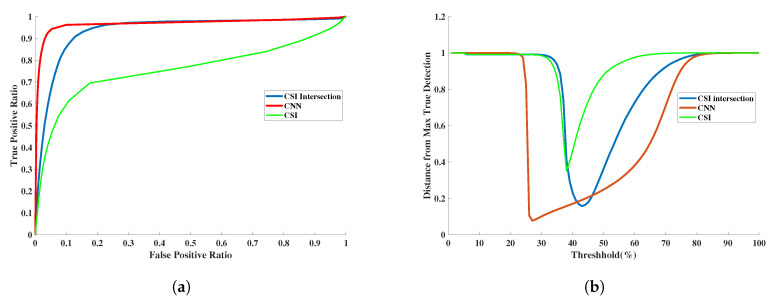
The detection performance using the reconstructed outputs of the Convolutional Neural Network (CNN) and CSI as well as the intersection of CSI reconstructions at the five chosen frequencies. (**a**) Receiver Operating Characteristics (ROC) curves derived from the reconstructions. (**b**) The DMTD curve.

The CNN-enhanced reconstruction performance and the subsequent tumor segmentation based on thresholding was evaluated quantitatively. The Root Mean Squared (RMS) reconstruction error between the network output and the true permittivity values was used to evaluate the reconstruction quality. The performance of a detection algorithm is often assessed in terms of two types of error i.e., False Positive Rate (FPR) and False Negative Rate (FNR). FPR and FNR will vary depending on the decision threshold used on the output score of the detection algorithm. To quantify the ability of the output score to separate the two classes, we need to analyze the two errors for all possible thresholds. In particular, we performed Receiver Operating Characteristics (ROC) analysis to assess the ability of the reconstructed complex permittivity to distinguish between tumor and non-tumor pixels. The ROC curve is a plot of True Positive Rate ($TPR=\frac{TP}{TP+FN}$) against the False Positive Rate ($FPR=\frac{FP}{FP+TN}$) for all thresholds. The Area Under the Curve (AUC) for the ROC is a metric quantifying the separability between tumor and non-tumor pixels [47]. For comparison we also computed RMS reconstruction error and performed ROC analysis on CSI-only reconstructions. ROC carries information about the relation of the true positives vs. the false positives. However, the information about the distributions of thresholds at which the different ratios fall would be lost in this curve. Therefore, the distance from any location on the ROC curve to the top-left corner of the plot is also an informative metric (we call this the “Distance-to-MaxTD” or “DMTD” plot). We use the DMTD curve as a complementary metric to display/analyze the relation between the true positive detection as well as the threshold at which a certain true positive to negative ratio happens. This will especially help us better understand the performance of the overlapping (or very similar) ROC curves for different scenarios. The depth of the curve tells us about the quality of the reconstruction; the lower the dip, the better the performance of the algorithm. The location of the dip carries information about the separation of the tumor relative to the background; for instance, the further the dip of the DMTD curve is to the left, the higher the separation between the background and tumor. Additionally, the width of the dip gives us information about the robustness of the algorithm; the wider the dip of the curve, the higher the chances of having a tumor with no artifacts (false positives) for the proper reconstruction of the tumor size and shape. The results of this quantitative evaluation by using four-fold cross-validation strategy for all 600 images are shown in Figure 3 and Table 2.

**Figure 4:**
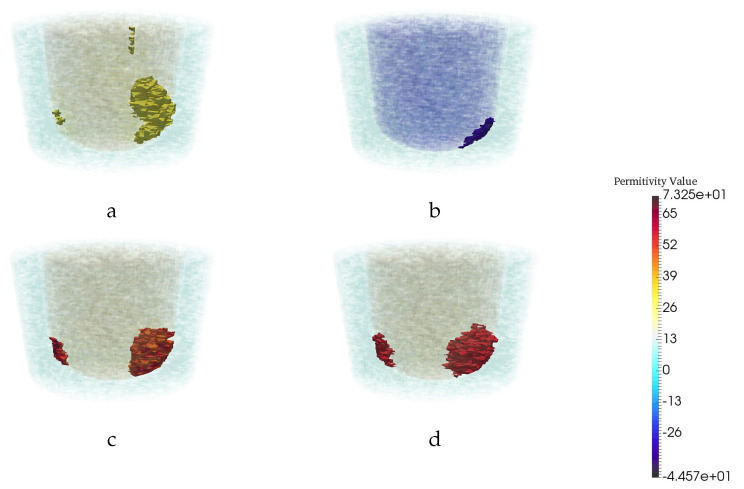
Reconstruction results for a particular example with two tumors. The real (**a**) and imaginary (**b**) part of CSI reconstruction at 1.1 GHz. (**c**) CNN reconstruction. (**d**) Ground truth.

**Figure 5:**
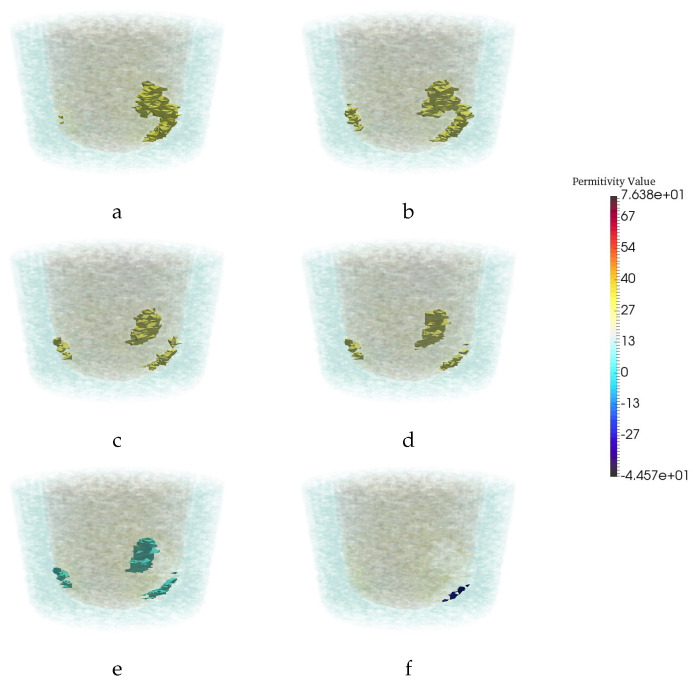
CSI reconstructions at four remaining frequencies for the same example as in Figure 4 and resulting images after intersecting images thresholded at 85% of the maximum reconstructed permittivity. (**a**–**d**) The real part of CSI reconstructions at 1.2, 1.3, 1.4, and 1.5 GHz. (**e**) Intersection of real part of CSI reconstructions. (**f**) Intersection of imaginary part of CSI reconstructions.

Figure 4 illustrates the performance of the trained U-Net in comparison with CSI reconstruction for an arbitrary example with two tumors. Based on the AUC and RMS error metrics, it could be concluded that the proposed CNN is successful in term of reconstruction and tumor detection. However, in a previous study [27], it was shown that taking the intersection of multi-frequency thresholded 3D images performs the best at detecting tumors. Therefore, we compared our trained CNN with the intersection of multi-frequency thresholded 3D images in terms of detection. The superiority of the trained CNN to CSI results as well as to multi-frequency thresholded results are shown in Figure 3. For this same example, the CSI reconstructions at the remaining four other frequencies are shown in Figure 5. The resulting images for the real and imaginary parts of the permittivity after taking the intersection of the reconstructions that were thresholded at 85% of the maximum reconstructed permittivity value are also shown in the figure. Note that results using a CNN trained to reconstruct the imaginary part of the complex permittivity (not shown) are very similar to those using the CNN trained to reconstruct the real part in terms of tumor detection (ROC Curve) and reconstruction performance (RMS error). Thus, the ROC curve in Figure 3 and RMS error in Table 2, were computed using only reconstructions of the real part of the permittivity.

### 3.4. Assessment of Robustness

It is important to assess the robustness of our trained neural network when being tested on images different from those used during training. We investigate four aspects of variation in test data as compared to the training data: (i) changes in frequencies used to generate CSI reconstructions, (ii) changes in breast phantom geometry, (iii) changes in prior-information, and (v) breast phantom with no tumor.

#### 3.4.1. Robustness to Changes in Frequency

**Figure 6:**
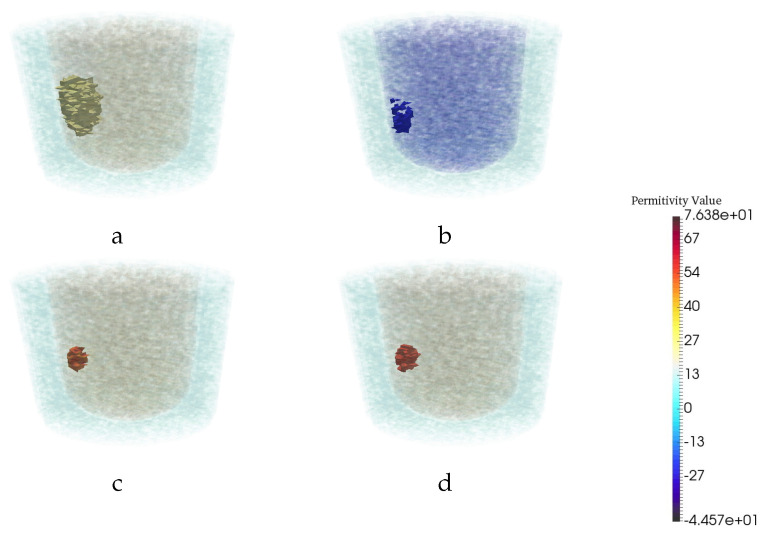
Reconstruction results for a particular example with one tumor at 1.05 GHz. The real (**a**) and imaginary (**b**) part of CSI reconstruction. (**c**) CNN reconstruction. (**d**) Ground truth.

First, given that the CNN was trained utilizing images created at 1.1 GHz, 1.2, 1.3, 1.4, and 1.5 GHz, the performance of the trained network was checked qualitatively by testing with CSI reconstructions that were created using data obtained at five arbitrarily chosen frequencies: 1.05, 1.15, 1.25, 1.35 and 1.45 GHz. Therefore, CSI reconstructions at chosen frequencies for five different breast phantoms have been created. These tests indicated that the trained U-Net was indeed superior to the CSI-only case. Results for one test example of the CSI and CNN outputs, from data obtained at 1.05 GHz, are shown in Figure 6. This suggests that the CNN is robust to testing images reconstructed using frequencies in the same bandwidth as used for training (one does not have to rely on using the exact same frequencies). As will be seen shortly, however, this is not the case once much higher frequencies are used. 

#### 3.4.2. Robustness to Changes in Breast Phantom Geometry

**Figure 7:**
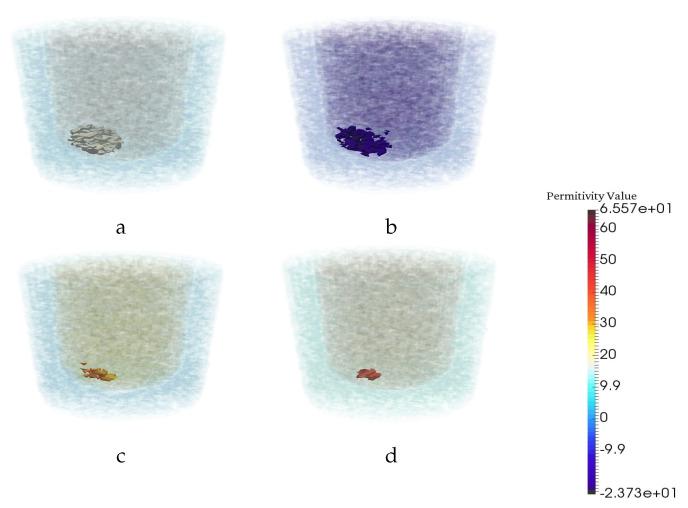
Reconstruction results for a particular example when the test images are CSI results for a breast phantom having a smaller fibroglandular region than those of the training set. The (**a**) real and (**b**) imaginary parts of the CSI reconstructions. (**c**) CNN reconstruction. (**d**) Ground truth.

The next test for the network’s robustess is to check against geometric changes of the breast phantom model. Thus, a new model which has the same dimensions for the fat region but has a smaller fibroglandular region (the height of fibroglandular region is decreased by 0.9 cm) was generated. By using this new small model, five different breast phantoms with a random tumor have been generated to evaluate the trained CNN. Figure 7 demonstrates the performance of the trained CNN for a particular example when the input images were CSI reconstructed images for this new model. As can be seen, the CNN significantly alters the CSI reconstructions (row 1) to bring them closer to the ground truth (row 2).

#### 3.4.3. Robustness to Imperfections in Prior Information

**Figure 8:**
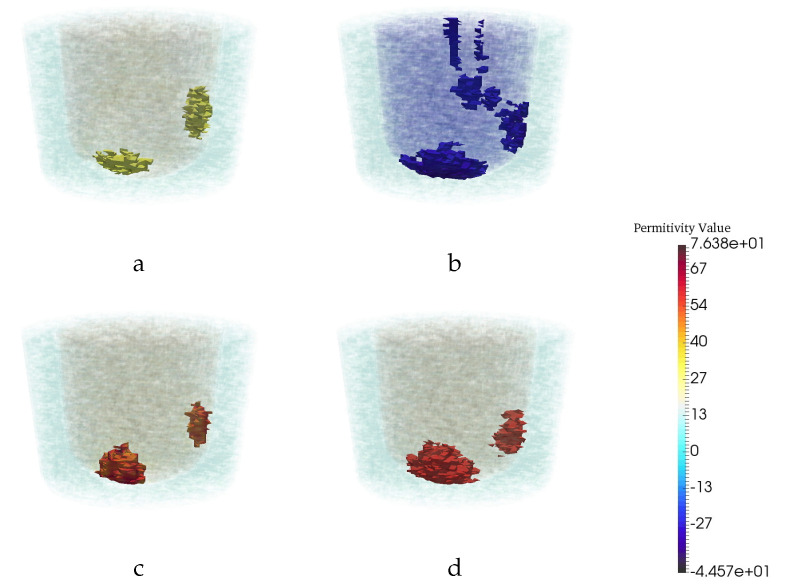
Reconstruction results for a particular example with two tumor when the training images are CSI results with perfect prior information, but the neural net was tested on imperfect prior information. The real (**a**) and imaginary (**b**) part of CSI reconstruction. (**c**) CNN reconstruction. (**d**) Ground truth.

**Figure 9:**
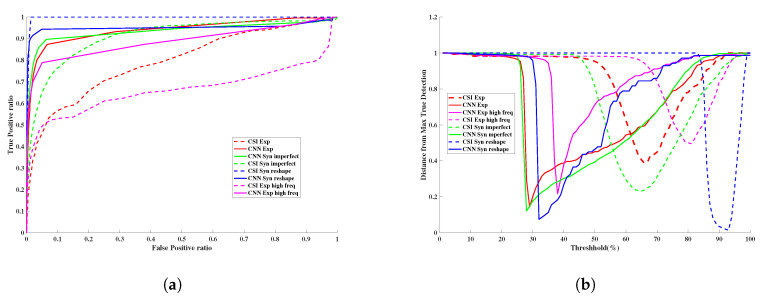
Detection performance based on the reconstructed outputs of CNN and CSI. (**a**) ROC curves derived from the reconstructed real part of the permittivity from CSI and CNN. (**b**) The DMTD. test cases are: synthetic: imperfect permittivity prior, and true breast phantom with elongated fibroglandular region. Experimental: using data within the frequency band and much higher than the training frequency band.

In order to understand the U-Net’s ability to remove artifacts, the next test case artificially induces artifacts into the CSI reconstructions by utilizing incorrect, or imperfect, prior information. Clearly, using perfect prior information results in very good CSI reconstructions; however, perfect prior information regarding the structural shape of the fibroglandular region as well as the permittivity of the fibroglandular tissue is difficult to obtain in practical circumstances. It is well known that using CSI with imperfect prior information produces various reconstruction artifacts. To evaluate this aspect of robustness we introduced 10% error in the permittivity of the fibroglandular tissue used as prior information. Figure 8 shows the performance of the CNN when tested with CSI reconstructions using imperfect permittivity in a structurally perfect fibroglandular region. The ROC curves created from the CSI and CNN outputs corresponding to this case shown in the plots of Figure 9. From the green colored curves we see that the CNN-enhanced reconstructions do provide an improvement over the CSI reconstructions. The distance-to-maxTD curve in Figure 9 clearly shows that the range in the threshold that could be used for good detection for the CNN-enhanced reconstructions is much wider than that could be used for the CSI reconstructions. When imperfect structural prior was used for a test case it was found that neither the CSI nor the CNN reconstructions performed well. This is the last test performed using synthetically generated images. 

#### 3.4.4. Robustness to Breast Phantom with No Tumor

**Figure 10:**
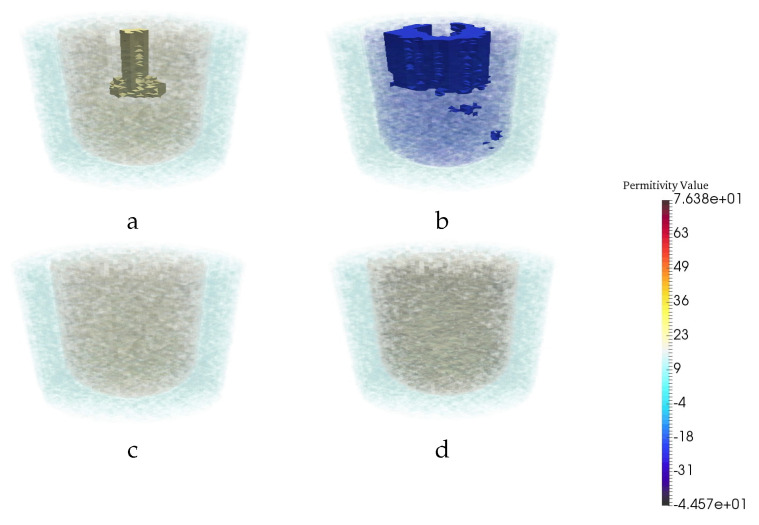
Reconstruction results for a particular example when the training images are CSI results with one or two tumors but the neural net was tested on a phantom with no tumor. The real (**a**) and imaginary (**b**) part of CSI reconstruction. (**c**) CNN reconstruction. (**d**) Ground truth.

Lastly, given that the CNN was trained only on breast phantom in presence of tumor, the last test in this section has been done to check the performance of the trained CNN for breast phantom with no tumor. Note that to prevent having zero scattered field data, we have to use imperfect prior information. We introduced 5% error in the permittivity of the fibroglandular tissue used as prior information. Figure 10 demonstrates the performance of the trained network when the input images were CSI reconstructed images with no tumor.

## 4. Experimental Tests and Results

**Figure 11:**
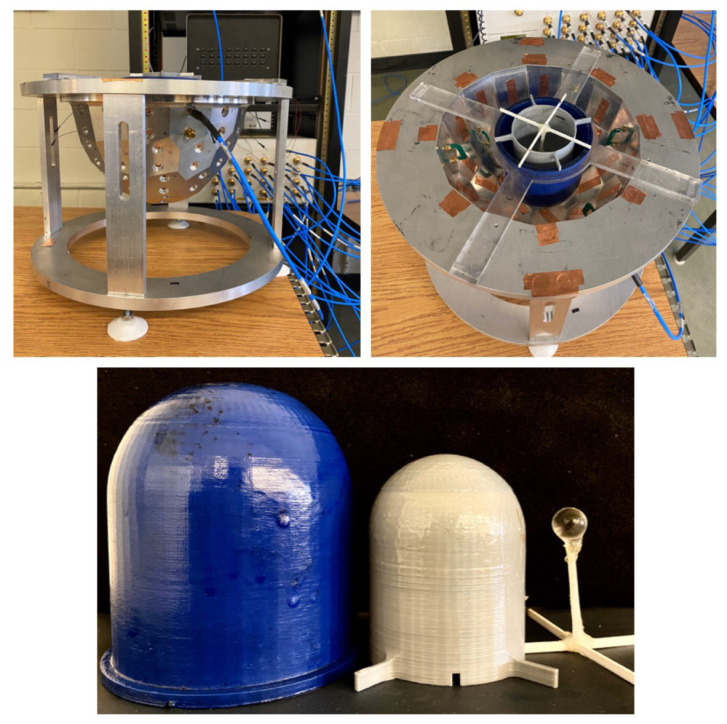
The experimental system including the three region breast phantom (Diameter of fat, fibroglanduar and tumor regions are 10, 8 and 2 CM respectively).

The experimental setup described in [27,42] was used to collect data to test the described neural network. A depiction of the imaging chamber and the breast phantom used in the experiment is shown in Figure 11. This chamber has 44 facets and contains 24 magnetic field probes and the breast phantom used in the chamber has three regions with similar sizes and properties to those of the numerical breast phantom described earlier for the fat and fibro regions; a 2 cm spherical phantom was used as the tumor region with properties similar to that of the tumor described in the numerical test cases. To mimic the properties close to those of a realistic breast, the fat region was filled with canola oil while a 20:80 ratio of water to glycerin is used to fill the fibroglanduar shell, and a 10:90 ratio of water to glycerin is used to fill the spherical inclusion representing a tumor. For these ratios, the permittivities of the canola oil and water/glycerin mixture are measured as 3.0 − j0.193, 23.3 − j18.1 and 50 − j25 respectively for fat, fibrogladular, and tumor at 1.1 GHz [27]. It is worth noting that this simplistic phantom is used as a simple proof of concept target for inverting a high contrast multilayered medium in an air background and not testing the system against realistic breast phantoms.

**Figure 12:**
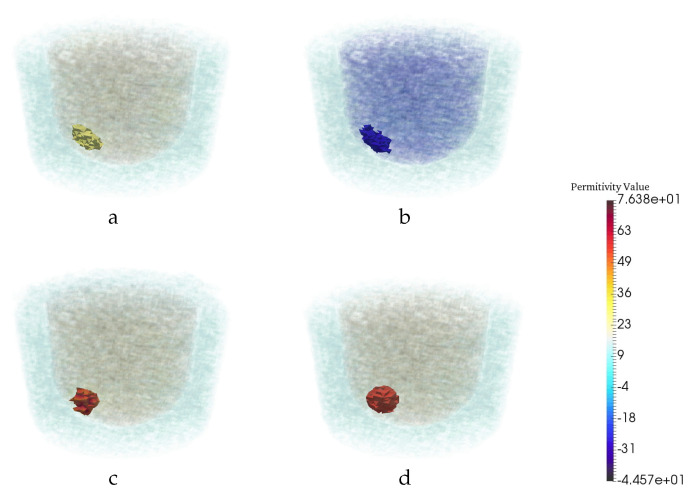
CNN performance for experimental result when the neural net was trained on Synthetic data. The real (**a**) and imaginary (**b**) part of CSI reconstruction. (**c**) CNN reconstruction. (**d**) Ground truth.

**Figure 13:**
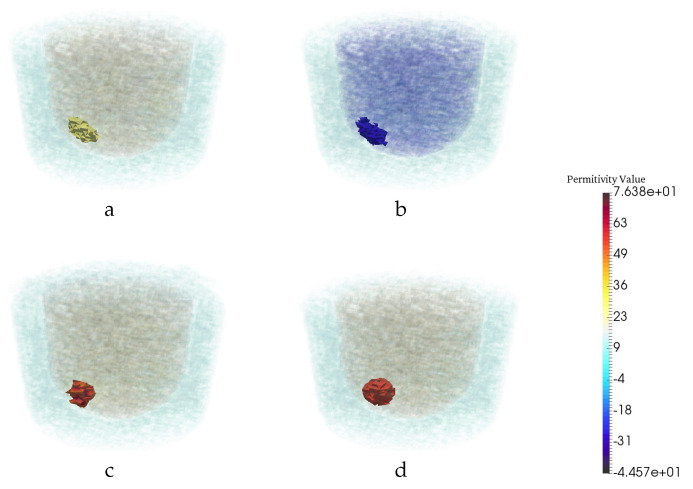
Reconstruction results for a particular example when the test images are CSI results in high frequencies but the neural net was trained on low frequencies. The real (**a**) and imaginary (**b**) part of CSI reconstruction. (**c**) CNN reconstruction. (**d**) Ground truth. (**e**) Intersection of real part of CSI reconstruction at all frequencies.(**f**) Intersection of imaginary part of CSI reconstruction at all frequencies(two intersection images are binary image).

In medical imaging, sometimes it is difficult to build a large experimental training data set. Therefore, it is desirable that a neural network trained on synthetic data generalizes well when tested on experimental data. To investigate this, we collected experimental data using a wide range of frequencies (1.1 to 2.9 GHz). The performance of the trained network for experimental data is evaluated and shown in Figure 12. Results illustrate that trained CNN improved the experimental CSI reconstructed images when frequencies similar or close to those for training data were used. However, when we tested the trained CNN with experimental images created with frequencies well beyond the band of frequencies used to create the training data, it is observed that CNN is not able to detect the tumor. Figure 13. One reason for this can be the significant change in the nature of the artifacts. In general, for the results presented in this manuscript, the artifacts at almost all lower frequency reconstructions have a lower permittivity compared to the value of the reconstructed tumor. However, the permittivities of the reconstructed artifacts at higher frequencies are higher than those of the reconstructed tumor.

## 5. Conclusions

A deep learning technique using a 3D CNN was developed to improve the imaging performance of 3D MWI of the breast. The improvement manifests as the removal of artifacts in the 3D reconstructions of the complex-valued permittivity of the breast being imaged. These reconstruction artifacts are specific to the MWI system wherein the microwave scattered-field data is collected as well as to the numerical inversion algorithm, in our case CSI, being used to create the images. Using synthetic 3D images that take both these factors into account, a CNN was trained with the goal to reproduce the true permittivity image of the breast from the artifact-laden 3D reconstructions. The trained CNN was tested with synthetic images as well as with images created using experimentally obtained microwave scattered-field data from an MWI system: the same MWI system for which a numerical model was utilized in the creation of the synthetic 3D images.

The RMS error between the CNN-reconstructed images and the true images are improved over the corresponding error between the CSI-only reconstructions and the true images. In addition, tumor detection was evaluated using ROC-AUC metrics and these are much improved for the CNN-reconstructed images over the ROC-AUC results for the CSI-only reconstructions. The results show that this deep learning technique has the ability to improve 3D CSI reconstructions in three interdependent ways. First, and foremost, the CNN has shown its ability to remove reconstruction artifacts which are a great challenge for quantitative MWI. Secondly, the trained CNN successfully corrects the permittivity values which tend to be undershot in the CSI reconstructions. Finally, from a qualitative perspective, the tumor location is more accurately reconstructed with respect to its true position and size.

There are several limitations of this work, but the most critical is that numerical phantoms with a single, relatively simple, fibroglandular region were utilized for training and testing. This same region was reproduced in the physical phantom utilized for the experimental results. Our experience with utilizing a similar technique with 2D images showed that this limitation can be removed by training with breast models having several types of fibroglandular regions. Similarly, this work has shown that when the artifacts are due to reconstructions obtained from data generated with MWI system parameters that were not utilized in the training set, for example artifacts generated by using microwave frequencies that are much higher than what the MWI system was designed for, then the trained CNN was not able to identify these as artifacts. In fact, some of these artifacts were identified as tumors. This result limits the robustness of the trained CNN but this study has provided a good understanding of that robustness. We further note that due to the significant level of computational resources required during the generation of forward data and inverse 3D CSI reconstructions, we generated only a moderately sized dataset consisting of 600 phantoms. Being aware of the limited number of training examples, we made extensive use of cross-validation and regularization techniques to avoid the possibility of model overfitting, which is evidenced by the generalization our CNN demonstrates on unseen examples. That said, having more training data would potentially help us to train a more robust CNN with better generalization properties. Techniques for overcoming some of these limitations will be investigated in planned future work.

## Figures and Tables

**Table 1:** Complex permittivity for different tissues.

Permittivity			
Air	Fat	Fibroglandular	Tumor
1 − 0.001j	3 − 0.6j	20 − 21.6j	56.3 − 30j

**Table 2:** Comparison of reconstruction and tumor detection performance.

	RMS Error		AUC	
	CSI	CNN	CSI	CNN
Synthetic Data	1.4356	1.161	0.935	0.957
Exprimental Data	1.250	1.172	0.794	0.938
